# Supplementary material for: The endoribonuclease Arlr is required to maintain lipid homeostasis by downregulating lipolytic genes during aging
Source: Nat Commun. 2023 Oct 6;14:6254. doi: 10.1038/s41467-023-42042-7 (PMC10558556; doi:10.1038/s41467-023-42042-7)
Supplement: Supplementary file 1 — Supplementary information [file 41467_2023_42042_MOESM1_ESM.pdf]

The endoribonuclease Arlr is required to maintain lipid homeostasis by downregulating lipolytic genes during aging

Xiaowei Sun[1], Jie Shen[1], Norbert Perrimon[2,3], Xue Kong[1] & Dan Wang[1*]

[1] Department of Plant Biosecurity and MARA Key Laboratory of Surveillance and Management for Plant Quarantine Pests, College of Plant Protection, China Agricultural University, Beijing, China.

[2] Department of Genetics, Blavatnik Institute, Harvard Medical School, Boston, MA, USA.

[3] Howard Hughes Medical Institute, Boston, MA, USA.

* Correspondence: dwang@cau.edu.cn

Supplementary information

This Supplementary information includes Supplementary Fig. 1–10 and Supplementary Table 1 and 2.

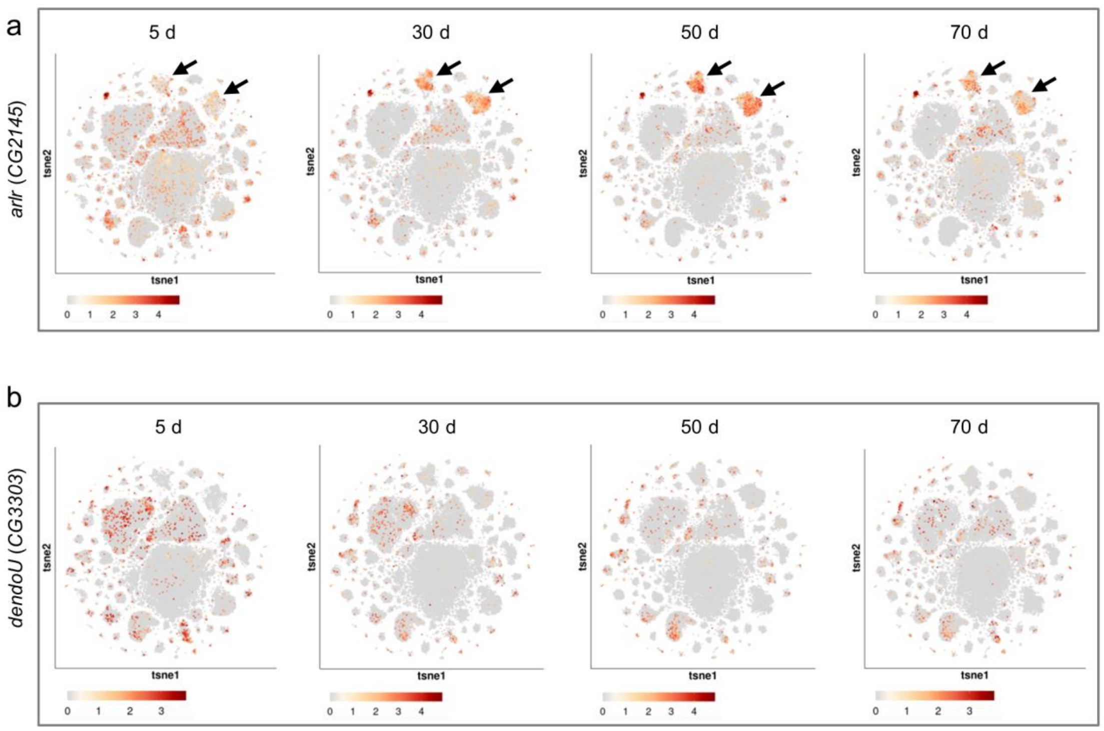

Supplementary Fig. 1. Single nuclei RNA sequencing expression of *arlr* and *dendoU* in the fat body during aging. a Expression of *arlr* was increased in aging flies. Arrows indicate the fat cell clusters. **b** Expression of *dendoU* did not change with age. d is short for days. Images are generated from data available at the Aging Fly Cell Atlas platform (https://hongjielilab.shinyapps.io/AFCA/).

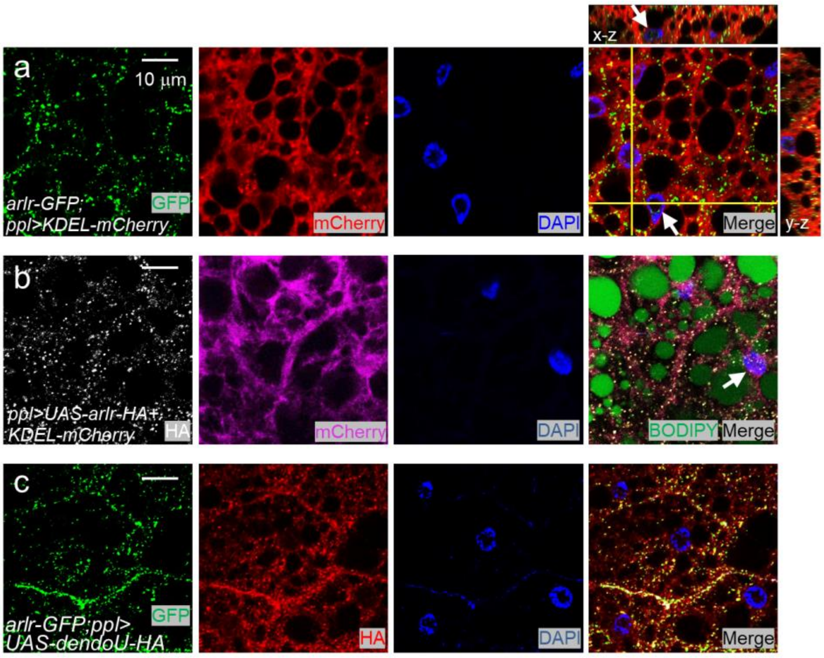

Supplementary Fig. 2. Arlr localizes to the ER. All panels in this figure are from females. **a** Co-expression of Arlr-GFP and the ER marker KDEL-mCherry at 1 week showing partial colocalization. The fusion protein Arlr-GFP labeled the endogenous expression of Arlr. Nuclei were stained with DAPI (blue). **b** HA staining to label Arlr. Arrows indicate small number of GFP puncta in the nuclei. **c** Co-staining of Arlr-GFP and DendoU-HA in the fat body. Scale bars are 10 µm.

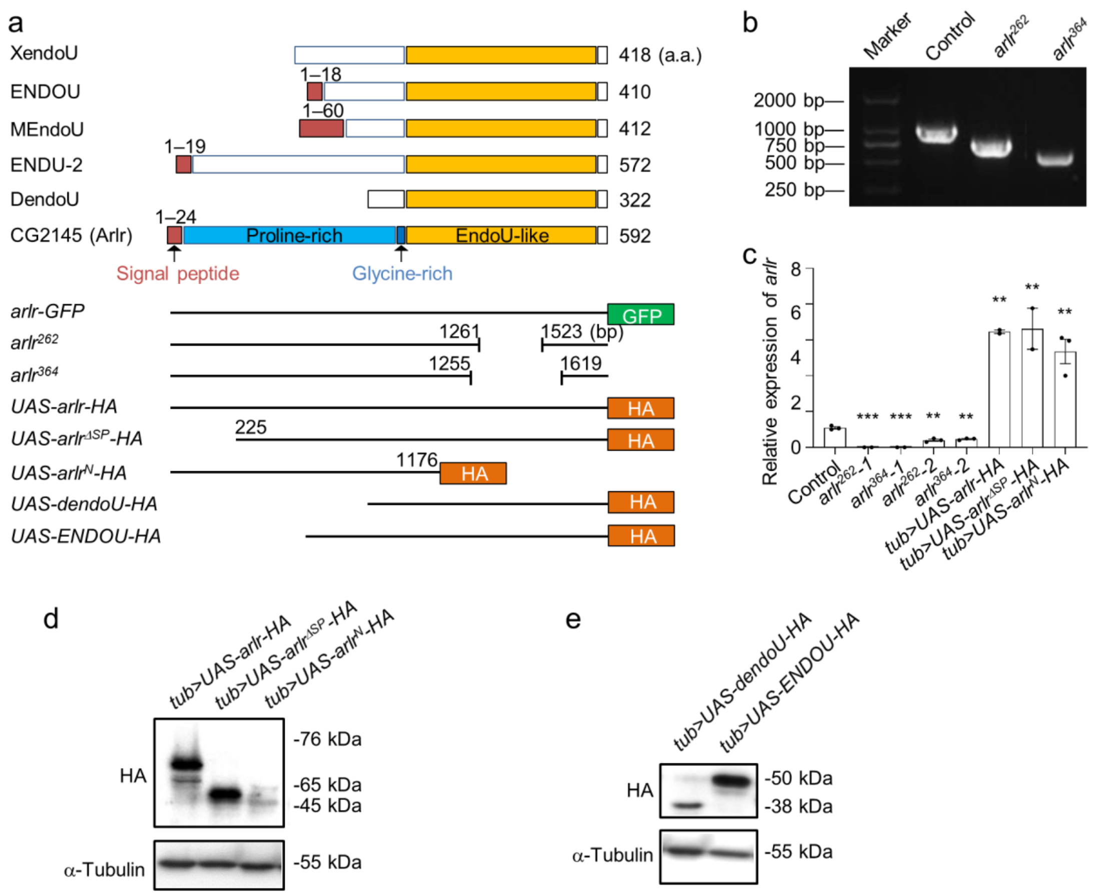

Supplementary Fig. 3. Validation of the genetic tools. a Structure of EndoU proteins and genetic tools generated in this study. The 1779 bp CDS of *arlr* encodes a 592 a.a.-length protein, which contains four domains: a signal peptide (1–24 a.a.), a proline-rich domain (43–294 a.a.), a glycine-rich domain (305–315 a.a.), and the EndoU-like domain (330–590 a.a.). The position of nucleotides in the CDS is indicated. **b** Genomic PCR results of control and *arlr* mutants (*arlr*^{262} and *arlr*^{364}). **c** qRT-PCR quantification of *arlr* mRNAs in different strains. A pair of primers flanking the deletion region of *arlr* mutants was designed for amplification in *arlr*^{262}*-1*, *arlr*^{364}*-1*, *tub>UAS-arlr-HA* and *tub>UAS-arlr*$^{\Delta SP}$*-HA* flies. Another pair of primers designed at the C terminus was used in *arlr*^{262}*-2*, *arlr*^{364}*-2*, and *tub>UAS-arlr*^{N}*-HA* flies. n=3 biologically independent experiments. Data were analyzed by one-way ANOVA with Tukey's multiple comparison test. *** $P < 0.001$; ** $P = 0.002$ for *arlr*^{262} and *arlr*^{364} mutants, ** $P = 0.003$ for *tub>UAS-arlr-HA* and *tub>UAS-arlr*$^{\Delta SP}$*-HA*, ** $P = 0.006$ for *tub>UAS-arlr*^{N}*-HA*. Error bars represent SEM. **d, e** Western blot showing the molecular weight of the various gene products.

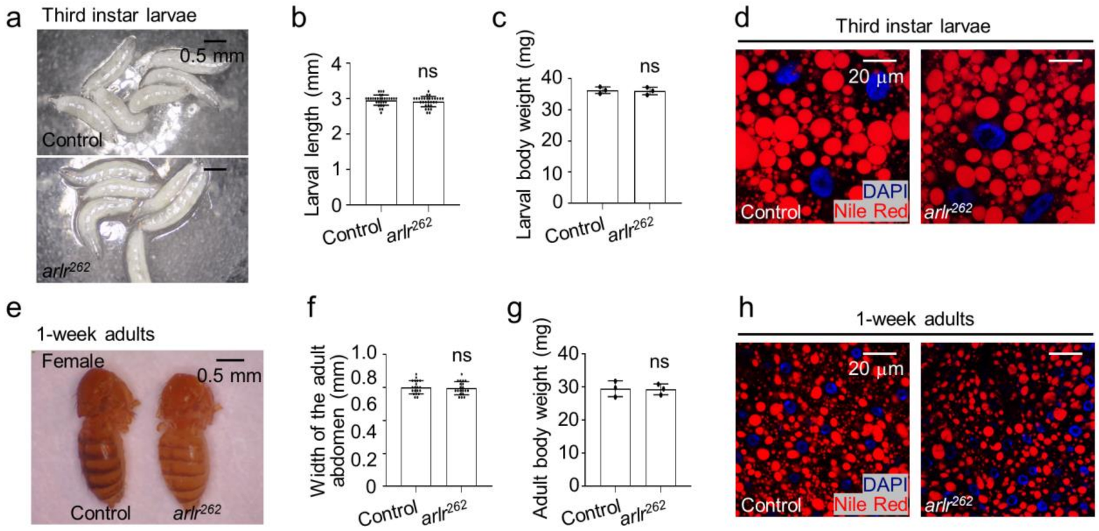

Supplementary Fig. 4. *arlr* mutants show no changes in body size and LD content during the larval and early adult stages. Larvae in panels a–d are from males and females. Panels e–h are from adult females. **a–c** *arlr* mutant third instar larvae are normal. Control and $arlr^{262}$ mutants larval length and weight are similar. n is from at least 20 biologically independent animals in b and n=3 biologically independent experiments in c. ns $P = 0.25$ in b and $P = 0.7908$ in c. **d** LDs revealed by Nile Red staining (red) in third instar larvae were similar in control and $arlr^{262}$ mutants. **e–g** One week old *arlr* adult mutants were normal in size. Quantification of the abdominal width (f) and weight (g) in control and $arlr^{262}$ mutants. n is from at least 20 biologically independent animals in f and n=3 biologically independent experiments in g. ns $P = 0.69$ in f and $P = 0.9114$ in g. **h** LDs in 1-week *arlr* mutants in control and $arlr^{262}$ mutants. Data were analyzed by independent two-sample *t* tests (two-tailed). Error bars represent SEM. Scale bars were 0.5 mm in the whole flies and 20 µm in dissected tissues.

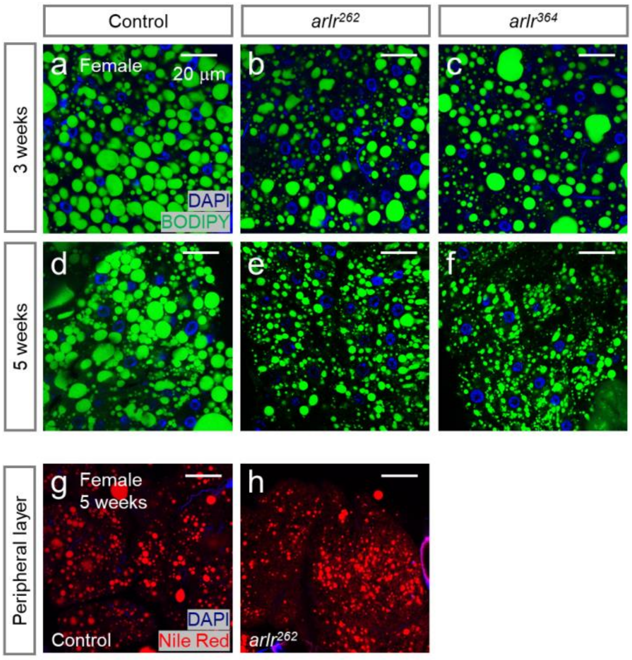

Supplementary Fig. 5. BODIPY staining and the peripheral layer of LDs. Panels are from adult females. **a**–**f** BODIPY staining (green) confirmed small LDs in *arlr* mutants. **g, h** LDs at the peripheral layer in the control and $arlr^{262}$ mutants. Both genotypes showed small LDs. Scale bars are 20 μm.

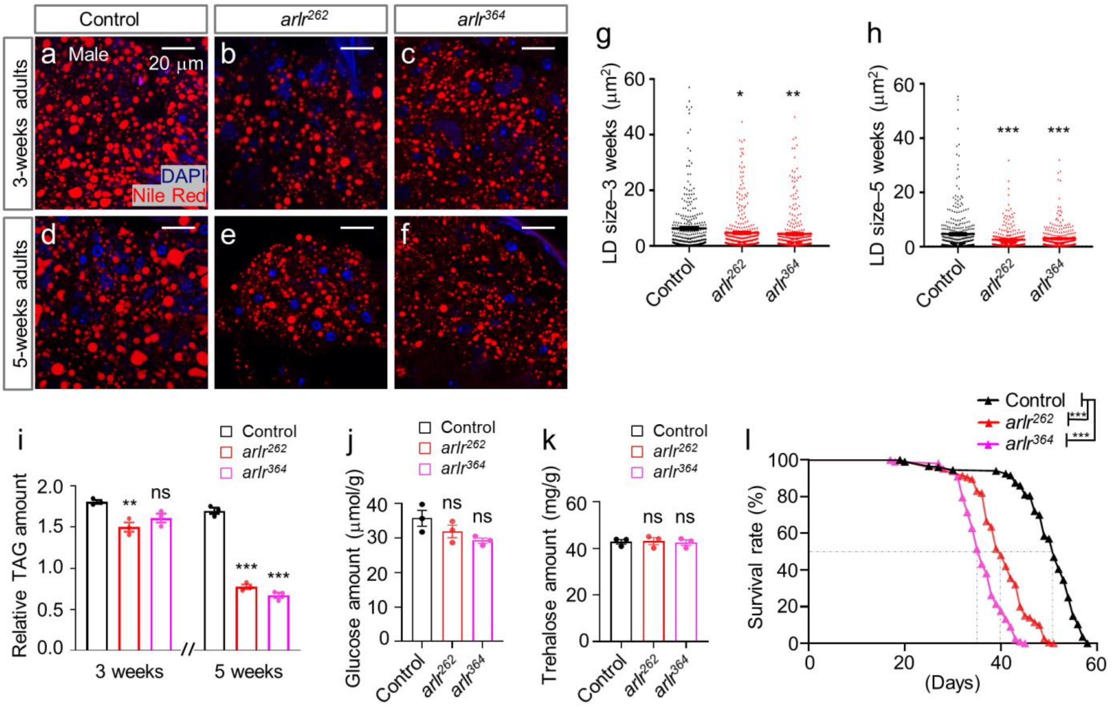

Supplementary Fig. 6. *arlr* male mutants show similar LD defects as the *arlr* female mutants. All panels in this Figure are from males. **a** LDs in control male fly at 3 weeks. **b, c** *arlr*^{262} and *arlr*^{364} mutant phenotypes at 3 weeks. **d** LD phenotype in control 5 weeks old flies. **e, f** *arlr*^{262} and *arlr*^{364} mutant phenotypes at 5 weeks. **g** Quantification of LD size at 3 weeks. * P = 0.034; ** P = 0.0046. **h** Quantification of LD size at 5 weeks. *** $P < 0.001$. n=6 biologically independent animals (3 samples in each animal) in g and h. **i** Relative TAG amount in control and mutant flies. ** P = 0.009; ns P = 0.06; *** $P < 0.001$. **j, k** Amount of whole body glucose and trehalose at 5 weeks. n=3 biologically independent experiments in i–k. ns P = 0.34 for *arlr*^{262} and P = 0.09 for *arlr*^{364} mutants in j; ns P = 0.99 for *arlr*^{262} and P = 0.98 for *arlr*^{364} mutants in k. **l** Lifespan of male flies. Dashed lines indicate the median lifespan, which was 51, 40 and 36 days in each of the three genotypes. *** $P < 0.001$. n=1 biologically independent experiment. Statistical data were analyzed by one-way ANOVA with Tukey's multiple comparison test in g–k and by Log-rank (Mantel-Cox) test in l. Error bars represent SEM. Scale bars in a–f are 20 μm.

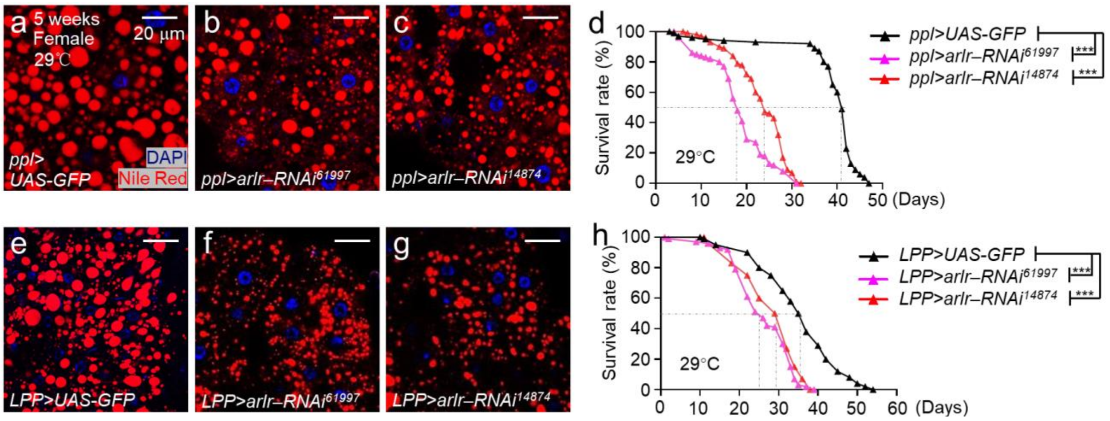

Supplementary Fig. 7. LD storage is reduced in aged *arlr*-knockdown flies. All panels in this Figure are from females. Flies are raised at 29 ℃. **a–c** Nile Red staining of LDs in the control and two independent *arlr* RNAi lines driven by ppl-Gal4 at 5 weeks. *ppl>UAS-GFP* is the control. *ppl>arlr–RNAi*^{61997} and *ppl>arlr–RNAi*^{14874} flies are associated with smaller LDs. **d** Lifespan was reduced in *arlr*-knockdown flies driven by ppl-Gal4. Dashed lines indicate the median lifespan, which was 41, 18 and 24 days in each of the three genotypes. *** $P < 0.001$. **e–h** *arlr* RNAi lines driven by LPP-Gal4 showed similar results with that driven by lpp-Gal4. Dashed lines in h indicate the median lifespan, which was 36, 26 and 30 days in each of the three genotypes. *** $P < 0.001$ in h. n=1 biologically independent experiment in d and h. Statistical data were analyzed by Log-rank (Mantel-Cox) test in d and h. Scale bars are 20 μm.

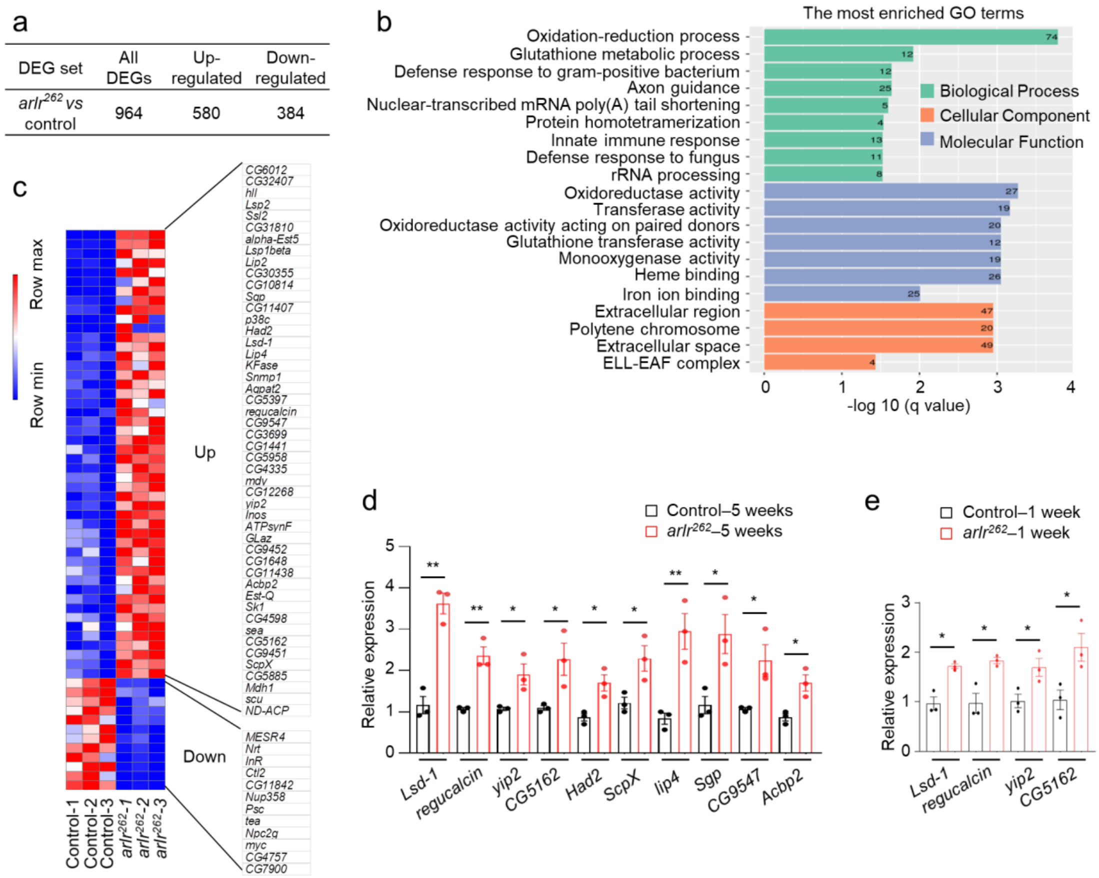

Supplementary Fig. 8. Arlr regulates the expression of lipid-associated genes. Adipose tissues attached with the abdominal cuticle from both males and females at 5 weeks were used in RNA-seq analysis. **a** Analysis of all DEGs in the transcriptome assay. **b** The most enriched GO terms of the DEGs. **c** Heat map of significantly changed genes in lipid metabolism. **d** qRT-PCR results confirmed the upregulation of lipid metabolism-associated genes in *arlr* mutants. The *P* value for each gene is ** *P* = 0.002, ** *P* = 0.004, * *P* = 0.03, * *P* = 0.04, * *P* = 0.02, * *P* = 0.03, ** *P* = 0.009, * *P* = 0.03, * *P* = 0.04, * *P* = 0.02, respectively. **e** qRT-PCR results of candidate genes at 1 week. n=3 biologically independent experiments in d and e. The *P* value for each gene is * *P* = 0.01, * *P* = 0.01, * *P* = 0.04, * *P* = 0.04, respectively. Data were analyzed by independent two-sample *t* tests (two-tailed). Error bars represent SEM.

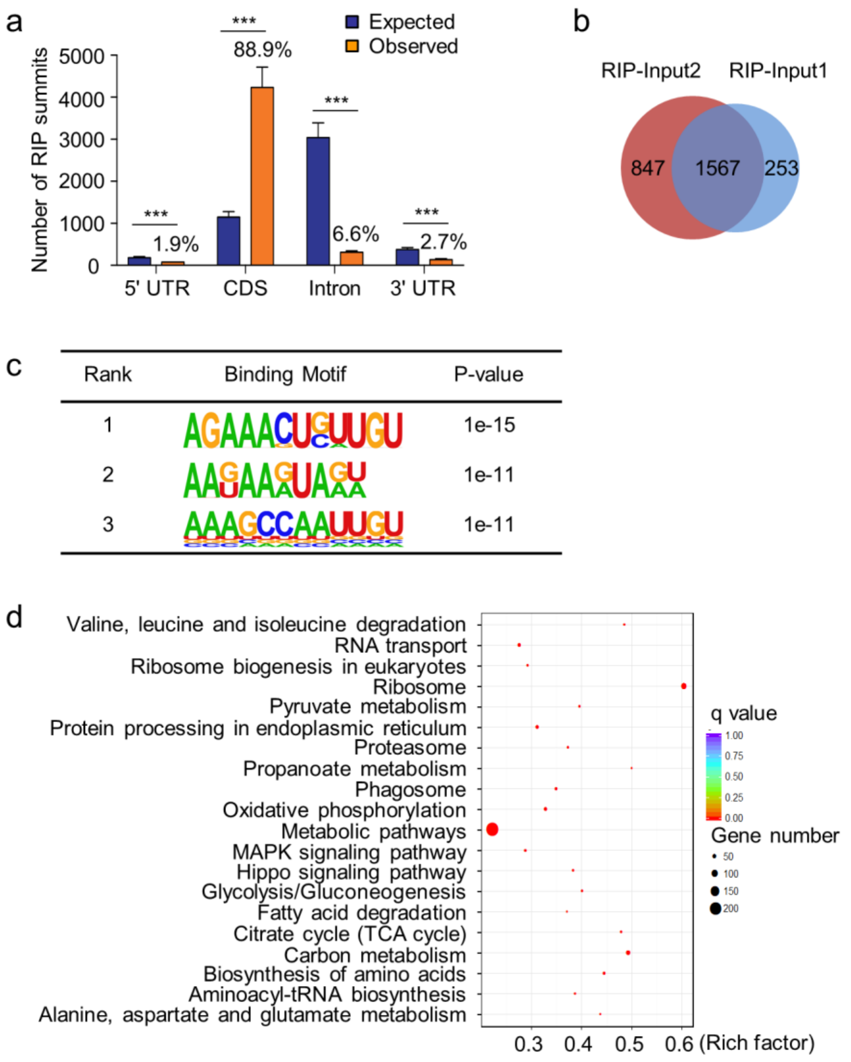

Supplementary Fig. 9. Arlr binds to target mRNAs. Adipose tissues attached with the abdominal cuticle at 5 weeks in both genders were used in this assay. **a** Expected and observed number of RIP submits (by plotting normalized reads) in the RIP-seq assay. **b** Number of genes identified in two independent RIP samples. **c** Predicted top three motifs in the peaks identified by Arlr. **d** KEGG enrichment of differential peaks.

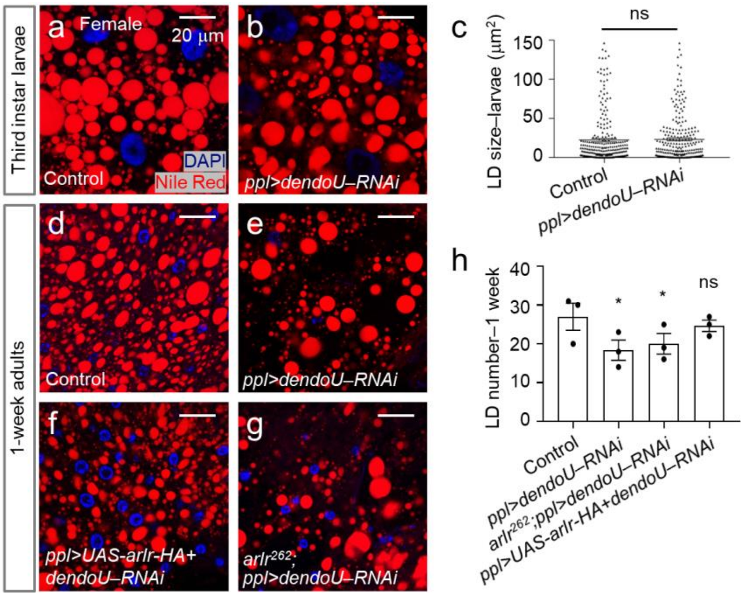

Supplementary Fig. 10. LD phenotype associated with *dendoU* by knockdown at 1 week. All panels in this Figure are from females. **a** LDs in control third instar larvae. **b** LDs in *dendoU* knockdown larvae were normal in size. **c** Quantification of LD size in panel a and b. n=6 biologically independent animals. ns P = 0.78. Data were analyzed by independent two-sample t tests (two-tailed). **d** LDs in 1-week adults in the control flies. **e** Knockdown of *dendoU* in 1-week adults showed fewer LDs. **f** Rescue of LD defects in *dendoU–RNAi* flies by *arlr*. **g** Reduction of both *arlr* and *dendoU* showed similar LD defects as *dendoU*-knockdown flies at 1 week. **h** Quantification of LD number per sample at 1 week. * P = 0.02; ns P = 0.87. n=3 biologically independent animals. Data were analyzed by Mixed-effects analysis Dunnett's multiple comparison test (two-tailed). Error bars represent SEM. Scale bars in a, b, and d–g are 20 μm.

Supplementary Table 1. Food ingredients.

Component (/L)	Standard food	Starvation diet	HSD	HFD	High nutrition diet	Poor nutrition diet
Agar (g)	5.33	5.33	5.33	5.33	5.33	20
Polenta (g)	62.16	/	62.16	62.16	74.6	6.216
Dry yeast (g)	24	/	24	24	28.8	2.4
White sugar (g)	62.5	/	437.5	62.5	75	6.25
p-Hydroxybenzoic acid (mL)	11.7	11.7	11.7	11.7	11.7	11.7
Coconut oil (mL)	/	/	/	100	/	/

Note: High sugar diet (HSD) is 7 times white sugar of the standard food and high fat diet (HFD) includes extra coconut oil. High nutrition diet contains 20% more polenta, dry yeast and white sugar than the amount in standard food, while poor nutrition diet contains 10% amount of the above three nutrients.

Supplementary Table 2. Primer sequences.

**Primers for generating *arlr* transgenic strains (5'–3')**	
UAS-arlr-HA-F	GGAATTCCGGCAAAGTGCTAATAAATCGAGC
UAS-arlr-HA-R	CTAGTCTAGACTAGTCAAGCGTAATCTGGAACATCGTATGGGTAAGCGTAATCTGGAACATCGTATGGGTAAGCGTAATCTGGAACATCGTATGGGTAAATCTCCGGATAGGCGCTG
UAS-arlr$^{\Delta SP}$-HA-F	GGAATTCCATGAAATCGGTGGTGGTTACACCC
UAS-arlr$^{\Delta SP}$-HA-R	CTAGTCTAGACTAGTCAAGCGTAATCTGGAACATCGTATGGGTAAGCGTAATCTGGAACATCGTATGGGTAAGCGTAATCTGGAACATCGTATGGGTAAATCTCCGGATAGGCGCTG
UAS-arlr^{N}-HA-F	GGAATTCC GGCAAAGTGCTAATAAATCGAGC
UAS-arlr^{N}-HA-R	CTAGTCTAGACTAGTCAAGCGTAATCTGGAACATCGTATGGGTAAGCGTAATCTGGAACATCGTATGGGTAAGCGTAATCTGGAACATCGTATGGGTACAGCCGCATCTTGACGAT
**Primers for PCR to test *arlr* mutants and transgenic strains (5'–3')**	
arlr mutant-F	GAGCAACAGCCAGATCGGTA
arlr mutant-R	GAAAGGCCCGCGAATTTGAA
UAS transgene-F	GCGCAGCTGAACAAGCTAAA
UAS transgene-R	AGCGTAGTCTGGGACGTCGTATGGGTA
arlr-GFP-F	GGGATCGTCACCTTTCGGCGCAT
arlr-GFP-R	GAGGAAGAATTCTGCTACATTTC

Primers for qRT-PCR (5'–3')	
arlr-F1	GTCAAGGAGCTGTGGTTCACAC
arlr-R1	TTATTGAGTCCCTGATGCGAGA
arlr-F2	TACGCCAAGGCAAAGTGCTA
arlr-R2	AGCCATTTCGCCTACCTGAC
dendoU-F	CTAACTCCACCACCGCTCTG
dendoU-R	ATATTGCTCCTGGACGTGCT
Lsd-1-F	ATGTTTGGCCACAAAAGCCC
Lsd-1-R	GAGCCTCTGCGATGGTTCTT
Had2-F	AGCGAGGGTCTGCAAAAGAG
Had2-R	AGCATTAGGTCACGGGTTCG
regucalcin-F	GTGGCTAGCAATCCCAAGGT
regucalcin-R	AGGTGGCCACATACAGGTTG
ScpX-F	GATGGCTTTAAGGTTGCGCC
ScpX-R	CTGTCCATTGGGTCCGTTGT
yip2-F	TGAAGGGCATCAACCAGACC
yip2-R	GAGGAGGACGCGATCACATT
lip4-F	GGCGTCGTTGATTGCACTTT
lip4-R	CGCCACGGATCTTCCCAAAA
CG5162-F	GATGGACCACCACCGTGAAT
CG5162-R	AAGCTTCACCAATCCGAGGG
Sgp-F	TCCATCACCTTTGGGAAGGC
Sgp-R	AAGGGCAGGTGGTAAAGTGG
CG9547-F	AATTCGGTCGTCCTTTGGCT
CG9547-R	GAAATCATGTCGGGCGTGTG
Acbp2-F	CGTCCCAGTGATGACGAGTT
Acbp2-R	TTGCCCTTCTGCTTGTTCCA
RPL32-F	ATGCTAAGCTGTCGCACAAATG
RPL32-R	GTTCGATCCGTAACCGATGT
actin-F	CAGAGCAAGCGTGGTATCCT
actin-R	CTCATTGTAGAAGGTGTGGTGC
RIP-Lsd-1-F	ATGATGTACTGGAACACCAA
RIP-Lsd-1-R	ATTCATTTCCGAAACCAATT
RIP-regucalcin-F	TCTCCAATGGACTGGCATGG
RIP-regucalcin-R	CAGGTTGCCCTCGGTATCGA
RIP-yip2-F	TCCTTTGTCGGAGTCAAGCC
RIP-yip2-R	ATCCAACTTCAGGGCATCGG
RIP-CG5162-F	GAGATGCCCACTTCGTCGAT
RIP-CG5162-R	GGGCACAGGTTACCGAGAAA
RIP-PGRP-LE-F	CTTACTCAAAACCGAAGAGATCG
RIP-PGRP-LE-R	CCTGGTGAATGATAGCTTACTCTG
RIP-iab-7-F	AGGAAGAGAGCGGAAAGTGCA
RIP-iab-7-R	CGGTCGCTCTTAGCCAATACTCTT
**Primers for luciferase reporter assay (5'–3')**	

Lsd-1-F	[illegible]GAGCTCGCAACTGCAACCAGCGG
Lsd-1-R	[illegible]CTCTAGAGTAGACGCCGTTGTATG
regucalcin-F	[illegible]GAGCTCTTACTGATTCCCGTGAT
regucalcin-R	[illegible]CTCTAGAGACCTTCAGGTTGACGC
CG5162-F	[illegible]GAGCTCGGACGAGTTCCGGCAAA
CG5162-R	[illegible]CTCTAGATCCTGAGAACCAGGAAC
yip2-F	[illegible]GAGCTCTCTGCCGCAACCAAAGG
yip2-R	[illegible]CTCTAGAGACAGCCTCGAGGAGAA
