# Supplementary material for: The endoribonuclease Arlr is required to maintain lipid homeostasis by downregulating lipolytic genes during aging
Source: Nat Commun. 2023 Oct 6;14:6254. doi: 10.1038/s41467-023-42042-7 (PMC10558556; doi:10.1038/s41467-023-42042-7)
Supplement: Supplementary file 3 — Description of Additional Supplementary Files [file 41467_2023_42042_MOESM3_ESM.pdf]

Description of Additional Supplementary Files

File Name: Supplementary Data 1
Description: Genes found in RNA-seq assay.

File Name: Supplementary Data 2
Description: Genes found in RIP-seq assay.
