# Supplementary material for: The endoribonuclease Arlr is required to maintain lipid homeostasis by downregulating lipolytic genes during aging
Source: Nat Commun. 2023 Oct 6;14:6254. doi: 10.1038/s41467-023-42042-7 (PMC10558556; doi:10.1038/s41467-023-42042-7)
Supplement: Supplementary file 6 — Reporting Summary [file 41467_2023_42042_MOESM6_ESM.pdf]

nature portfolio

Corresponding author(s): Dan Wang
Last updated by author(s): Sep 8, 2023

Reporting Summary

Nature Portfolio wishes to improve the reproducibility of the work that we publish. This form provides structure for consistency and transparency in reporting. For further information on Nature Portfolio policies, see our Editorial Policies and the Editorial Policy Checklist.

Statistics

For all statistical analyses, confirm that the following items are present in the figure legend, table legend, main text, or Methods section.

n/a	Confirmed	
☐	☒	The exact sample size (*n*) for each experimental group/condition, given as a discrete number and unit of measurement
☐	☒	A statement on whether measurements were taken from distinct samples or whether the same sample was measured repeatedly
☐	☒	The statistical test(s) used AND whether they are one- or two-sided<br>*Only common tests should be described solely by name; describe more complex techniques in the Methods section.*
☒	☐	A description of all covariates tested
☒	☐	A description of any assumptions or corrections, such as tests of normality and adjustment for multiple comparisons
☐	☒	A full description of the statistical parameters including central tendency (e.g. means) or other basic estimates (e.g. regression coefficient) AND variation (e.g. standard deviation) or associated estimates of uncertainty (e.g. confidence intervals)
☐	☒	For null hypothesis testing, the test statistic (e.g. *F*, *t*, *r*) with confidence intervals, effect sizes, degrees of freedom and *P* value noted<br>*Give P values as exact values whenever suitable.*
☒	☐	For Bayesian analysis, information on the choice of priors and Markov chain Monte Carlo settings
☒	☐	For hierarchical and complex designs, identification of the appropriate level for tests and full reporting of outcomes
☒	☐	Estimates of effect sizes (e.g. Cohen's *d*, Pearson's *r*), indicating how they were calculated

Our web collection on statistics for biologists contains articles on many of the points above.

Software and code

Policy information about availability of computer code

Data collection	For immunostaining, Zeiss LSM 800 confocal microscope was used to get images. RNA-seq and RIP-seq were performed using Illumina novaseq 6000 platform (Illumina). qRT-PCR was performed using the QuantStudio 6 Flex platform. Luciferase reporter assay results were tested by SpectraMax i3x (TECAN, Switzerland).
Data analysis	GraphPad Prism 9.5 was used to generate the statistical charts. Images were processed with Photoshop 2021. In the RIP-seq assay, the Integrative Genomics Viewer (IGV) software (http://software.broadinstitute.org/software/igv/) was used for genomic loci analysis. Potential motifs in the target sequences were predicted using Homer software (V4.11, University of California at San Diego, http://homer.ucsd.edu/homer/). Image J V1.53 was used to measure the area of LDs.

For manuscripts utilizing custom algorithms or software that are central to the research but not yet described in published literature, software must be made available to editors and reviewers. We strongly encourage code deposition in a community repository (e.g. GitHub). See the Nature Portfolio guidelines for submitting code & software for further information.

Data

Policy information about availability of data

All manuscripts must include a data availability statement. This statement should provide the following information, where applicable:

- Accession codes, unique identifiers, or web links for publicly available datasets
- A description of any restrictions on data availability
- For clinical datasets or third party data, please ensure that the statement adheres to our policy

The RNA-seq data generated in this study have been deposited in the Genbank database under accession code PRJNA943130 [https://www.ncbi.nlm.nih.gov/bioproject/PRJNA943130]. The ChIP-seq data generated in this study have been deposited in the Genbank database under accession code PRJNA943378 [https://www.ncbi.nlm.nih.gov/bioproject/PRJNA943378]. Gene expression images are available at the Aging Fly Cell Atlas platform [https://hongjielilab.shinyapps.io/AFCA/]. Source data are provided with this paper.

Human research participants

Policy information about studies involving human research participants and Sex and Gender in Research.

Reporting on sex and gender	not applicable
Population characteristics	not applicable
Recruitment	not applicable
Ethics oversight	not applicable

Note that full information on the approval of the study protocol must also be provided in the manuscript.

Field-specific reporting

Please select the one below that is the best fit for your research. If you are not sure, read the appropriate sections before making your selection.

☒ Life sciences ☐ Behavioural & social sciences ☐ Ecological, evolutionary & environmental sciences

For a reference copy of the document with all sections, see nature.com/documents/nr-reporting-summary-flat.pdf

Life sciences study design

All studies must disclose on these points even when the disclosure is negative.

Sample size	No sample size calculation was performed in this study.<br>For RNA-seq and RIP-seq, 20 individual adults (n=20) were used in each replicate. For lifespan measurements, at least 100 flies in total in each replicate (n>100) was used. For triglyceride, glucose and trehalose measurements, 10 adult flies (n=10) were used in each replicate. To determine the LD phenotype, at least 10 adults were dissected and imaged, and quantification was performed using 6 flies, 3 adipocytes in each (n=6). The total number of LD in this sample size is moderate to show in the figures and meets the requirement of minimum replicates for statistical analysis.
Data exclusions	No data were excluded.
Replication	All findings were replicated. For qRT-PCR assay, triglyceride, glucose and trehalose measurements, three biological replicates in each genotype were performed at a time. For lipid droplet phenotype, 10 flies were dissected at a time and repeated later. For lifespan, the second measurement was performed after the last one.
Randomization	All samples were allocated randomly.
Blinding	The investigators were blinded to group allocation during data collection and/or analysis.

Reporting for specific materials, systems and methods

We require information from authors about some types of materials, experimental systems and methods used in many studies. Here, indicate whether each material, system or method listed is relevant to your study. If you are not sure if a list item applies to your research, read the appropriate section before selecting a response.

Materials & experimental systems

n/a	Involved in the study
☐	☒ Antibodies
☐	☒ Eukaryotic cell lines
☒	☐ Palaeontology and archaeology
☐	☒ Animals and other organisms
☒	☐ Clinical data
☒	☐ Dual use research of concern

Methods

n/a	Involved in the study
☐	☒ ChIP-seq
☒	☐ Flow cytometry
☒	☐ MRI-based neuroimaging

Antibodies

Antibodies used	Primary antibodies: rabbit anti-GFP (A11122, Invitrogen) , HA-Tag (C29F4) rabbit mAb (#3724, Cell Signaling), and mouse monoclonal anti-α-tubulin (1:50,000, Sigma, T6074). Secondary antibodies: goat anti-rabbit Alexa 647 (A21245, ThermoFisher) , goat anti-rabbit Alexa 488 (A21206, ThermoFisher), goat anti-mouse IgG (H+L), and HRP (1:20,000, BE0102, Easybio, Beijing, China).
Validation	Rabbit anti-GFP: https://www.thermofisher.cn/cn/zh/antibody/product/GFP-Antibody-Polyclonal/A-11122.<br>HA tag: https://www.cellsignal.cn/products/primary-antibodies/ha-tag-c29f4-rabbit-mab/3724?site-search-type=Products&N=4294956287&Ntt=%233724&fromPage=plp&_requestid=89828.<br>Anti-α-tubulin: https://www.sigmaaldrich.cn/CN/zh/product/sigma/t6074.

Eukaryotic cell lines

Policy information about cell lines and Sex and Gender in Research

Cell line source(s)	The HEK293T cell line was purchased from ATCC (USA) and maintained in our lab.
Authentication	The cell line was STR tested.
Mycoplasma contamination	The cell line was tested negative for mycoplasma contamination.
Commonly misidentified lines (See ICLAC register)	No.

Animals and other research organisms

Policy information about studies involving animals; ARRIVE guidelines recommended for reporting animal research, and Sex and Gender in Research

Laboratory animals	Drosophila melanogaster. The fly strains from BDSC, VDRC, and THFC were purchased since 2018 to now. UAS-KDEL-mCherry is a gift from Yixian Cui (Wuhan University, Wuhan, China) in 2021. The arlr gene related flies generated in this study were in 2019. It's about 10 days per generation and adult flies can live for about three months at 25°C.
Wild animals	The study did not involve wild animals.
Reporting on sex	This information has not been collected.
Field-collected samples	The study did not involve samples collected from the field.
Ethics oversight	No ethical approval or guidance was required because Drosophila melanogaster is an insect.

Note that full information on the approval of the study protocol must also be provided in the manuscript.

ChIP-seq

Data deposition

☒ Confirm that both raw and final processed data have been deposited in a public database such as GEO.

☒ Confirm that you have deposited or provided access to graph files (e.g. BED files) for the called peaks.

Data access links<br>*May remain private before publication.*	https://www.ncbi.nlm.nih.gov/bioproject/PRJNA943378
Files in database submission	GFP_IP1_R1.fq.gz<br>GFP_IP1_R2.fq.gz<br>GFP_IP2_R1.fq.gz<br>GFP_IP2_R2.fq.gz

March 2021

	GFP_Inp1_R1.fq.gz<br>GFP_Inp1_R2.fq.gz<br>GFP_Inp2_R1.fq.gz<br>GFP_Inp2_R2.fq.gz
Genome browser session (e.g. UCSC)	not applicable

Methodology

Replicates	Two replicates for Input and IP groups, respectively.
Sequencing depth	The total number of reads is 22006988, 23518184, 32897791 and 24432832 in Input1, Input2, IP1 and IP2, respectively. The uniquely mapped reads are 2656539, 2638550, 4841906 and 3425128 in Input1, Input2, IP1 and IP2, respectively. The average length of reads is 158, 145, 230 and 215 in Input1, Input2, IP1 and IP2, respectively. All reads are paired.
Antibodies	rabbit anti-GFP (A11122, Invitrogen)
Peak calling parameters	After mapping reads to the reference Drosophila genome, we used MACS2 (version 2.1.0) peak calling software to identify regions of IP enrichment over background. A q-value threshold of 0.05 was used in all data sets. After peak calling, the distribution of chromosome distribution, peak width, fold enrichment, significant level and peak summit number per peak were all displayed.
Data quality	Raw data (raw reads) of fastq format are processed using fastp software. Clean data (clean reads) are obtained by removing reads containing adapter, reads containing ploy-N and low-quality reads from raw data. At the same time, Q20, Q30 and GC content of the clean data are calculated. All the downstream analyses are based on the clean data with high quality.
Software	The fragment distribution and concentration of RNAs (after immunoprecipitation) were tested using Agilent 2100 bioanalyzer (Agilent) and simpliNano spectrophotometer (GE Healthcare). Immunoprecipitated RNA and Input control were used for library construction by NEB Next® Ultra™ RNA Library Prep Kit (New England Biolabs). Library quality was assessed on the Agilent Bioanalyzer 2100 system. The libraries were sequenced on an Illumina Novaseq platform with a paired-end read length of 150 bp according to standard protocols. Sequencing was carried out with two independent biological replicates.<br>Index of the reference genome was built using BWA (v 0.7.12) and clean reads were aligned to the reference genome using BWA mem (v 0.7.12).<br>MACS2 (version 2.1.0) peak calling software was used to identify peak regions of IP enrichment over background.<br>Homer (v4.9.1) was used to detect denovo sequence motifs and matched known motifs.
